# Clinical comparison between direct anterior approach and posterior lateral approach in total hip arthroplasty and risk factors for lateral femoral cutaneous nerve injury

**DOI:** 10.3389/fsurg.2025.1482731

**Published:** 2025-04-01

**Authors:** Jian-feng Yan, Le Zhao, Qiang Li

**Affiliations:** Department of Orthopedics, The Affiliated Changshu Hospital of Nantong University, Changshu No. 2 People’s Hospital, Changshu, Jiangsu, China

**Keywords:** total hip arthroplasty, direct anterior approach, posterior lateral approach, lateral femoral cutaneous nerve injury, predictive model, efficacy assessment

## Abstract

**Objective:**

This study aimed to compare the clinical outcomes of total hip arthroplasty (THA) using the lateral decubitus direct anterior approach (DAA) vs. the traditional posterior lateral approach (PLA), and to explore the risk factors and predictive models for lateral femoral cutaneous nerve (LFCN) injury following DAA-THA.

**Methods:**

Two hundred patients undergoing primary unilateral THA were randomly assigned to the DAA group and the PLA group, with 100 cases in each group. Clinical data, surgical parameters, postoperative pain scores, and other relevant data were recorded, and the differences between the two groups in terms of treatment outcomes and postoperative recovery were analyzed.

**Results:**

Compared to the PLA group, patients in the DAA group had shorter incisions, reduced blood loss and drainage, shorter hospital stays, decreased postoperative inflammatory markers, and lower pain scores. However, the incidence of postoperative LFCN injury was higher in the DAA (DAA:24patients, 24%, PLA:2patients, 2%) group, although most cases resolved within one year. Univariate analysis showed that diabetes, preoperative serum CK levels, serum IL-6, and TNF-α levels at postoperative day 3 were associated with early LFCN injury, while diabetes, BMI, and postoperative inflammation were significantly associated with persistent LFCN injury. Binary logistic regression analysis identified serum IL-6 and TNF-α levels at postoperative day 3 as independent risk factors for persistent LFCN injury. The established predictive model demonstrated good discrimination.

**Conclusion:**

Despite slightly longer surgical duration, DAA demonstrated significant advantages in reducing early pain, blood loss, and hospital stay. However, postoperative LFCN injury warrants attention, especially in patients with diabetes and postoperative inflammatory reactions.

## 1 Background

Hip disorders, such as necrosis of the femoral head and osteoarthritis, often affect the elderly, resulting in hip pain and limited mobility, which seriously affects quality of life (1). Total hip replacement is the primary procedure for the treatment of hip disorders (e.g., fracture of the femoral neck, necrosis of the femoral head, osteoarthritis) (2). Total hip replacement is a major surgical breakthrough in removing damaged tissue and replacing it with an artificial prosthesis to relieve pain and improve function. The artificial prosthesis consists of acetabular and femoral components, and technological advances have made the materials more durable, extending the life of the prosthesis (3, 4).

Currently, the choice of surgical approach for THA includes the traditional Posterior Lateral Approach (PLA) and the relatively newer Direct Anterior Approach (DAA) (5). The traditional PLA technique, matured over many years, improves surgical visibility, facilitating exposure of the proximal femur and acetabulum. However, during surgery, significant trauma and bleeding occur as muscles such as the piriformis, superior and inferior gemellus, obturator externus, and part of the quadratus femoris need to be cut and disrupted. Postoperative pain is pronounced, and recovery is slow, increasing the occurrence of complications such as late hip joint dislocation and deep vein thrombosis (6–9).

With the increasing demand for the treatment of hip joint diseases, minimally invasive surgery and rapid recovery have become urgent needs for many patients. In recent years, DAA-THA, as an outstanding representative of minimally invasive surgery, has attracted widespread attention and interest from orthopedic surgeons worldwide. The DAA approach, which does not require cutting any muscles, directly exposes the hip joint through muscle gaps (10), maintaining the integrity of the soft tissues around the hip joint, reducing surgical trauma, and facilitating early recovery of hip joint function (11, 12).

However, DAA-THA surgery also presents challenges. Firstly, compared to PLA, DAA surgery is more complex and requires a longer learning curve, necessitating surgeons to possess extensive anatomical knowledge and surgical experience (13). Secondly, the incidence of lateral femoral cutaneous nerve (LFCN) injury during DAA surgery is high, around 30%, and may be due to traction, laceration, or mistaken ligation (14). Therefore, this study aims to compare the clinical outcomes of DAA and PLA approaches in THA and explore the risk factors for LFCN injury after DAA-THA to enhance patient postoperative satisfaction and quality of life.

## 2 Materials and methods

### 2.1 Study design and data collection

This was a prospective study, which was approved by the Medical Ethics Committee of Changshu Second People's Hospital, and all patients gave informed consent. All the patients were operated on by the same surgeon. And we used intraoperative imaging.A total of 200 patients from Changshu Second People's Hospital, undergoing unilateral total hip arthroplasty (THA) for the first time between July 1, 2021, and June 30, 2023, were selected. Patients were randomly assigned into the Direct Anterior Approach (DAA) group or the Posterior Lateral Approach (PLA) group based on admission order, with odd-numbered patients assigned to the DAA group and even-numbered patients to the PLA group.

Inclusion criteria: (a) patients who needed to undergo total hip arthroplasty; (b) patients who underwent total hip arthroplasty for the first time; (c) patients with normal preoperative coagulation index tests; (d) patients with biologic-type prostheses used in the operation (15); (e) patients who did not receive autologous or allogeneic blood transfusion during the perioperative period; and (f) patients who had signed the informed consent for the operation (16, 17).

Exclusion criteria: (a) patients with necrotic or relatively weakened hip abductor muscle strength; (b) patients with a previous history of hip surgery; (c) severe osteoporosis; (d) severe acetabular dysplasia (Crowe types III and IV); (e) stiff hips; (f) pathological fracture; (g) patients with disease comorbidities that severely affect postoperative recovery (e.g., polio and Parkinson's disease); and h) patients with poor compliance who were unable to co-operate with or patients who fail to follow up. Surgical Procedure: The DAA group underwent lateral decubitus DAA total hip arthroplasty, while the PLA group underwent lateral decubitus posterior lateral approach total hip arthroplasty. Preoperative routine examinations and preparations were conducted, with cefuroxime used for infection prophylaxis during surgery. Postoperative pain relief, anti-inflammatory treatment, and physical therapy were administered to prevent deep vein thrombosis. Postoperative Clinical Indicators: Comparative analysis of postoperative pain levels, recovery rates, and complication rates between the two groups were conducted. The clinical efficacy of DAA and PLA in total hip arthroplasty and the risk factors for postoperative LFCN injury were evaluated to provide surgeons with more accurate surgical choices and postoperative management strategies, thus improving patient quality of life (18).

### 2.2 Clinical data and laboratory tests

This study aimed to compare the application of lateral decubitus DAA and PLA in THA and postoperative recovery, exploring the clinical efficacy of THA-DAA in the treatment of hip joint diseases. Therefore, relevant clinical indicators were meticulously recorded and observed.

#### 2.2.1 General data

Patient demographics: Including age, gender, BMI index, smoking and drinking history, ASA classification, comorbidities, and etiology.

### 2.3 Clinical data

#### 2.3.1 Surgical technical index assessment

The Nakata method was used to evaluate the position of the femoral stem, while the Pradhan method was used to measure the abduction angle, anteversion angle of the acetabular prosthesis, and the internal and external rotation angles of the femoral stem.

#### 2.3.2 Clinical functional assessment

The Harris scoring system was used to evaluate hip joint function preoperatively and postoperatively (at 2 weeks, 4 weeks, and 6 months postoperatively) in both groups of patients. This scoring system assesses hip joint function from six aspects: pain, function, mobility, strength, deformity, and stability, with scores compared between the two groups of patients.

#### 2.3.3 Pain assessment

The Visual Analog Scale (VAS) was used to measure hip pain levels in patients preoperatively and at 24, 48, and 72 h postoperatively, quantifying subjective pain perception.

#### 2.3.4 Adverse event recording

Detailed records of intraoperative fractures, LFCN injuries, early dislocations, prosthetic loosening, infections, joint stiffness, and lower limb thrombosis were maintained, along with the management methods for each complication.

### 2.4 Laboratory tests

#### 2.4.1 Surgical index recording

Including surgical incision length, intraoperative blood loss, postoperative drainage volume, etc., to evaluate surgical difficulty and postoperative bleeding.

#### 2.4.2 Blood index monitoring

Recorded the decrease in hemoglobin (Hb) concentration 1 day postoperatively, as well as the serum levels of CK, CRP, IL-6, IL-1β, and TNF-α immediately postoperatively and at 3 days postoperatively. These indicators reflect the degree of postoperative inflammatory response and tissue damage, aiding in the assessment of surgical safety and postoperative recovery.

### 2.5 Analysis of risk factors

Lasso-Logistic regression was employed to screen independent risk factors for LFCN injury in patients undergoing DAA total hip arthroplasty. The occurrence of LFCN injury after surgery was used as the outcome indicator. Univariate analysis was conducted to screen variables, followed by multifactorial Logistic regression to determine the final variables and analyze the risk factors affecting LFCN injury in patients undergoing DAA total hip arthroplasty.

### 2.6 Model construction

Multifactorial Logistic regression was utilized to screen important variables, and the “rms” package in R software was employed to construct a predictive model for early LFCN injury in patients undergoing DAA total hip arthroplasty, presented in the form of a nomogram. Simultaneously, peripheral blood biochemical indicators at immediate and postoperative day 3 were utilized for diagnostic analysis of LFCN injury. The diagnostic value of each individual indicator and their combinations was evaluated through ROC curves, while the goodness of fit of the predictive model was assessed via Hosmer and Lemeshow tests.

### 2.7 Data analysis

Statistical analysis was performed using SPSS 23.00. Differences in count data between groups were assessed using the chi-square test. Continuous data are presented as mean ± standard deviation. Normally distributed data were analyzed using the *t*-test, while non-normally distributed data were analyzed using the Mann-Whitney *U*-test. Pearson correlation (for normally distributed data) and Spearman correlation (for non-normally distributed data) were used for correlation analysis. Logistic regression was employed to screen risk factors, ROC curves were used to evaluate diagnostic value, and ridge regression was used for multivariate regression. A significance level of *P* < 0.05 was considered significant. The Hosmer and Lemeshow tests were used to assess model fit, with *P* > 0.05 indicating a good fit. The significance level was set at *α* = 0.05.

## 3 Results

### 3.1 General data analysis

This study included a total of 200 patients, with 100 cases in the DAA group. The mean age was 63.17 ± 9.92 years, with an equal gender distribution, and a BMI of 24.94 ± 3.64 kg/m^2^. In the PLA group, there were also 100 cases, with a mean age of 62.88 ± 9.20 years, a slightly different gender distribution, and a BMI of 24.12 ± 3.55 kg/m^2^. There were no statistically significant differences between the two groups in terms of age, gender, or BMI (*P* > 0.5). There were no significant differences between the two groups regarding smoking or drinking history (*P* > 0.5). The incidence of hypertension, diabetes, and hyperlipidemia also did not differ significantly between the two groups (*P* > 0.5). ASA scores and the etiology of replacement surgery showed no significant differences between the two groups (*P* > 0.5), as shown in Table 1.

**Table 1:** General characteristics analysis of patients in the DAA and PLA groups.

Variables		DAA group (*n* = 100)	PLA group (*n* = 100)	*χ*2/t	*P* value
Gender (female/male)		50/50	45/55	0.132	0.479
Age (years)		63.17 ± 9.92	62.88 ± 9.20	0.214	0.830
BMI (kg/m^2^)		24.94 ± 3.64	24.12 ± 3.55	1.163	0.109
Smoking history (yes/no)		18/82	29/71	1.287	0.095
Alcohol consumption history (yes/no)		26/74	27/73	0.009	1.000
Hypertension (yes/no)		23/77	25/75	0.042	0.741
Diabetes (yes/no)		13/87	12/88	0.046	0.831
Hyperlipidemia (yes/no)		6/94	7/93	0.082	0.774
ASA classification		2.02 ± 0.60	2.08 ± 0.53	−0.795	0.454
Disease diagnosis	Osteoarthritis	34	30		
	Femoral head necrosis	33	38	0.623	0.891
	Congenital hip dislocation	9	9		
	Femoral neck fracture	24	23		

### 3.2 Surgical procedures and comparison of surgical characteristics

**Figure 1:**
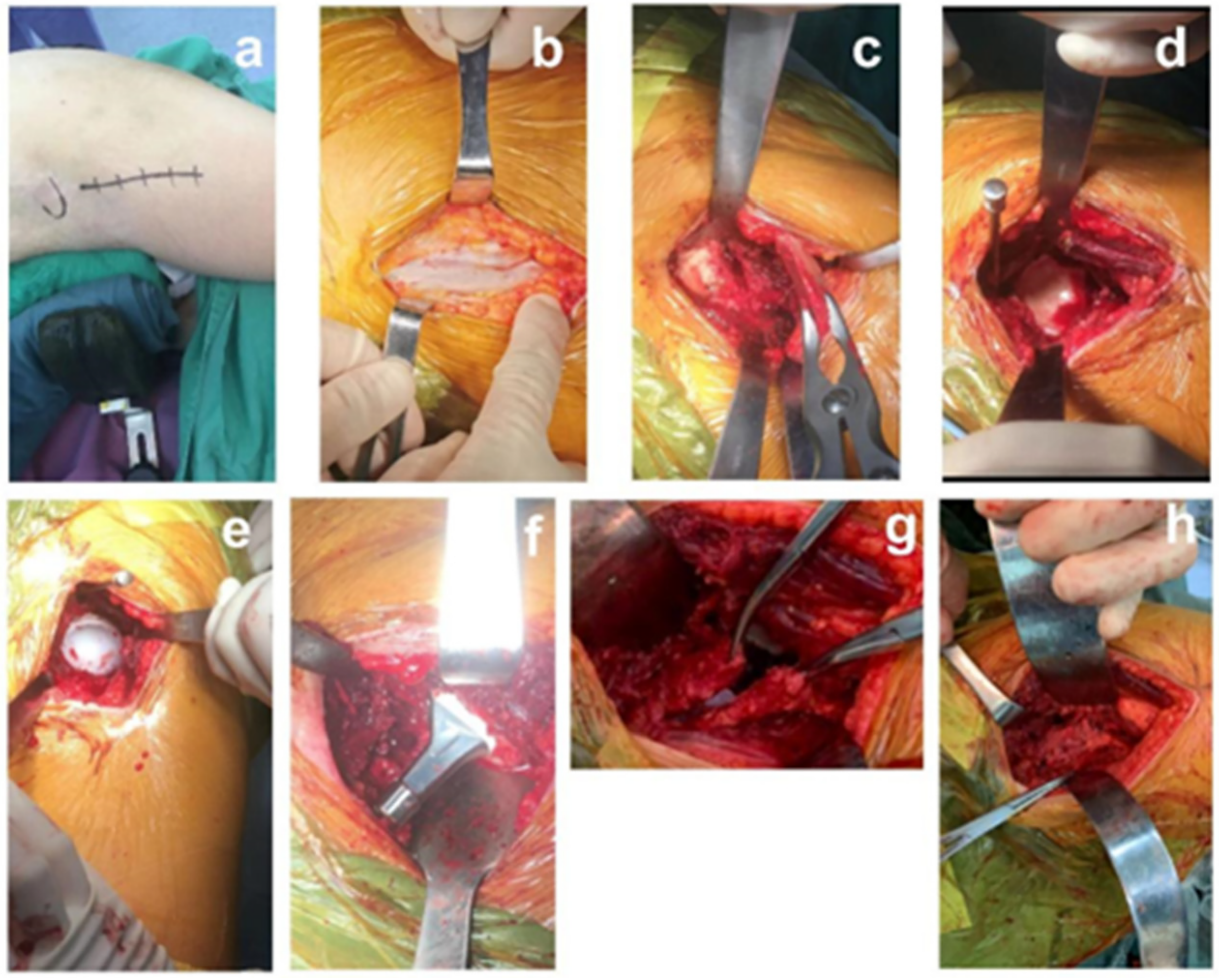
Schematic diagram of DAA total hip arthroplasty in lateral position.

Brief description of the operation in DAA group: the patient was anaesthetized and lying on the side, disinfected and toweled, and the operation was performed as usual. The incision was started below the lateral aspect of the anterior superior iliac spine, extended towards the fibular tuberosity, and peeled layer by layer until the muscle gap was exposed. The muscle was retracted using Hohamnn and S pull hooks to expose the Hueter's gap, and the ascending branch of the rotary lateral femoral artery was isolated and ligated. After incision of the joint capsule, the femoral head was removed by osteotomy. The acetabulum was exposed, hyperplastic tissue was removed, and the cup and liner were filed and placed. The joint capsule was processed, the proximal femur was extremely externally rotated, the medullary cavity was opened, and the femoral stem and head prosthesis were installed. Repositioning and inspection, cleaning and suturing, placement of drains and closure of the incision. The specific procedure is shown in Figure 1.

In the DAA group, the length of surgical incision was 7.40 ± 0.51 cm, the length of hospital stay was 7.22 ± 0.89 days, intraoperative blood loss was 118.99 ± 29.96 ml, the duration of surgery was 80.14 ± 8.51 min, and the hemoglobin concentration on the first postoperative day was 11.5 ± 1.44 g/dl. In the PLA group, the length of surgical incision was 10.34 ± 0.78 cm, the length of hospital stay was 8.77 ± 1.37 days, intraoperative blood loss was 184.68 ± 50.14 ml, the duration of surgery was 74.24 ± 10.36 min, and the hemoglobin concentration on the first postoperative day was 16.3 ± 1.55 g/dl. The length of surgical incision in the DAA group was significantly shorter (*P* = 0.010), with reduced intraoperative blood loss and postoperative drainage volume, and significantly shorter hospital stay (*P* < 0.001), but the duration of surgery was longer than that in the PLA group (*P* < 0.001), as shown in Table 2.

**Table 2:** Analysis of surgical characteristics in patients undergoing direct anterior approach (DAA) and posterior lateral approach (PLA).

Indicator	DAA group (*n* = 100)	PLA group (*n* = 100)	T value	*P* value
Surgical incision length (cm)	7.40 ± 0.51	10.34 ± 0.78	−31.547	<0.001
Length of hospital stay (d)	7.22 ± 0.89	8.77 ± 1.37	−9.488	<0.001
Intraoperative blood loss (ml)	118.99 ± 29.96	184.68 ± 50.14	−11.247	<0.001
Duration of surgery (min)	80.14 ± 8.51	74.24 ± 10.36	4.401	<0.001
Hemoglobin concentration loss on postoperative day 1 (g/dl)	11.5 ± 1.44	16.3 ± 1.55	8.235	<0.001

### 3.3 Analysis of serum levels of CK, CRP, Il-6, Il-1β, and TNF-α in both groups

Statistical analysis was conducted on the levels of CK, CRP, IL-6, IL-1β, and TNF-α in the peripheral blood of patients immediately postoperatively and on postoperative day 3 in both groups. The results showed no significant difference in the levels of CK, CRP, IL-6, IL-1β, and TNF-α in the peripheral blood immediately postoperatively between the two groups (*P* > 0.05). However, the levels of CK, CRP, IL-6, IL-1β, and TNF-α in the peripheral blood on postoperative day 3 were significantly lower in the DAA group compared to the PLA group (*P* < 0.05), as shown in Table 3.

**Table 3:** Analysis of serum levels of CK, CRP, IL-6, IL-1β, and TNF-α in the DAA and PLA groups.

Group		DAA group (*n* = 100)	PLA group (*n* = 100)	T value	*P* value
	Preoperative	117.91 ± 9.50	117.96 ± 7.98	0.043	0.970
CK (U/L)	Postoperative immediate	224.70 ± 13.04	251.22 ± 14.32	−13.693	<0.001
	Postoperative day 3	344.24 ± 23.39	402.28 ± 23.6	−17.468	<0.001
	Preoperative	4.84 ± 2.91	4.18 ± 2.63	1.683	0.094
CPR (mg/L)	Postoperative immediate	74.35 ± 29.44	88.37 ± 26.46	−3.542	<0.001
	Postoperative day 3	59.89 ± 28.92	76.61 ± 26.49	−4.263	<0.001
	Preoperative	101.63 ± 21.53	107.23 ± 23.05	−1.776	0.078
IL-6 (pg/ml)	Postoperative immediate	175.09 ± 28.41	215.32 ± 41.19	−8.040	<0.001
	Postoperative day 3	325.24 ± 74.51	382.94 ± 46.56	−6.567	<0.001
	Preoperative	27.39 ± 3.45	26.60 ± 3.15	1.691	0.091
IL-1β (pg/ml)	Postoperative immediate	47.51 ± 5.44	54.53 ± 6.89	−7.997	<0.001
	Postoperative day 3	73.43 ± 7.91	91.00 ± 9.30	−14.391	<0.001
	Preoperative	24.61 ± 14.58	26.57 ± 13.31	−0.993	0.320
TNF-α (pg/L)	Postoperative immediate	63.89 ± 21.76	79.74 ± 25.40	−4.739	<0.001
	Postoperative day 3	80.33 ± 26.29	92.88 ± 25.65	−3.417	0.001

### 3.4 Positioning of artificial acetabular prosthesis after DAA and PLA total hip arthroplasty

A comparative analysis was conducted on the anteversion angle, abduction angle, and varus/valgus angle of the artificial acetabular prosthesis, as well as the internal and external rotation angles of the femoral stem, in patients from the DAA and PLA groups. By comparing the postoperative abduction angle, acetabular anterior tilt angle, neck rod angle, angle between prosthesis stem and femoral axis, length difference between both lower limbs and eccentric distance between the two groups, it was found that the differences in each index between the DAA group and the PLA group after surgery were not statistically significant (*P* > 0.05), and the specific information is shown in Table 4.

**Table 4:** Abduction angle, anteversion angle, neck-shaft angle, angle between prosthesis stem and femoral axis, difference in length between lower limbs, and eccentric distance of acetabular prostheses in DAA and PLA groups.

Group	DAA group (*n* = 100)	PLA group (*n* = 100)	T value	*P* value
Abduction angle (°)	37.37 ± 3.70	37.30 ± 3.52	0.137	0.891
Anteversion angle (°)	17.66 ± 4.10	17.76 ± 4.50	−0.164	0.875
Neck-shaft angle (°)	134.36 ± 2.66	133.78 ± 2.63	1.550	0.123
Angle between prosthesis stem and femoral Axis (°)	0.79 ± 0.45	0.77 ± 0.43	0.321	0.689
Difference in length between lower limbs (mm)	6.53 ± 2.17	6.46 ± 1.99	0.238	0.810
Eccentric distance (mm)	42.44 ± 2.99	42.51 ± 2.39	−0.183	0.853

### 3.5 Comparison of preoperative and postoperative VAS pain scores in DAA and PLA patients

The VAS scoring system was used to assess hip pain in patients both before and after surgery at 24 h, 48 h, and 72 h. The results showed that the postoperative VAS scores were significantly lower than the preoperative scores at all time points in both groups (*P* < 0.05). Furthermore, the VAS scores at 24 h, 48 h, and 72 h postoperatively in the DAA group were significantly better than those in the PLA group (*P* < 0.05). However, there was no statistically significant difference in VAS scores at 2 weeks postoperatively (*P* > 0.05), as shown in Table 5.

**Table 5:** Comparison of VAS scores between direct anterior approach (DAA) and posterior lateral approach (PLA).

Time Points	DAA group (*n* = 100)	PLA group (*n* = 100)	χ2/t	*P* value
Preoperative	4.93 ± 2.05	4.84 ± 2.13	0.304	0.761
Postoperative 24 h	1.58 ± 1.05	2.55 ± 1.42	−5.493	<0.001
Postoperative 48 h	1.29 ± 1.08	2.21 ± 1.37	−5.274	<0.001
Postoperative 72 h	1.10 ± 0.97	1.53 ± 1.28	−2.677	0.008

### 3.6 Analysis of Harris scores for clinical function of the hip joint in DAA and PLA groups

Patients in both groups were followed up, and the Harris scores for clinical function of the hip joint at 2 weeks, 4 weeks, and 6 months postoperatively were as Table 6. The Harris scores in both groups significantly improved postoperatively compared to preoperative scores (*P* < 0.05). The Harris scores in the DAA group at 2 weeks and 4 weeks postoperatively were significantly better than those in the PLA group (*P* < 0.05). However, there was no statistically significant difference between the two groups at 6 months postoperatively (*P* > 0.05). Refer to Table 6 for details.

**Table 6:** Comparison of harris scores at different time points postoperatively between the DAA and PLA groups.

Time Points	DAA group (*n* = 100)	PLA group (*n* = 100)	χ^2^/t	*P* value
Postoperative	50.60 ± 8.52	50.70 ± 10.34	−0.075	0.941
Postoperative 2 weeks	77.78 ± 8.92	74.62 ± 10.53	2.290	0.023
Postoperative 4 weeks	85.44 ± 8.22	80.22 ± 11.06	3.788	0.000
Postoperative 6 months	89.79 ± 8.13	89.02 ± 9.96	0.599	0.550

### 3.7 Analysis of intraoperative and postoperative complications in patients undergoing total hip arthroplasty with DAA and PLA approaches

A one-month follow-up was conducted for both groups of patients, and postoperative complications were recorded and analyzed. In the DAA group, there were 2 cases of intraoperative fractures, 1 case of prosthetic loosening, 1 case of joint stiffness, and 3 cases of lower limb thrombosis. In the PLA group, there was 1 case of intraoperative fracture, 1 case of early dislocation, 1 case of prosthetic loosening, 1 case of infection, 1 case of joint stiffness, and 5 cases of lower limb ischemia. None of the aforementioned complications showed significant statistical differences between the two groups (*P* > 0.05). However, there was a significant difference in the occurrence of LFCN injuries between the DAA group (24 cases) and the PLA group (2 cases) (*P* < 0.001), as shown in Table 7.

**Table 7:** Analysis of complications in patients undergoing total hip arthroplasty via the direct anterior approach (DAA) and the posterior lateral approach (PLA).

Group	DAA (*n* = 100)	PLA group (*n* = 100)	χ^2^	*P* value
Intraoperative fracture	2	1	0.038	0.561
LFCN injury	24	0	27.272	<0.001
Early dislocation	0	1	1.005	0.316
Prosthetic loosening	1	1	0.000	1.000
Infection	0	1	1.005	0.316
Joint stiffness	1	1	0.000	1.000
Lower limb thrombosis	3	5	0.521	0.470

### 3.8 Single-factor analysis of risk factors for early and persistent LFCN injury

Statistically processed data indicate that single-factor analysis shows no significant statistical significance (*P* > 0.05) in the relationship between age, gender, disease diagnosis, surgical duration, ASA classification, incision length, intraoperative blood loss, postoperative drainage volume, and postoperative hemoglobin concentration at one day with the occurrence of postoperative LFCN injury symptoms (Table 8). However, diabetes status, preoperative serum creatine kinase (CK) levels, postoperative serum interleukin-6 (IL-6) levels at three days, and tumor necrosis factor-alpha (TNF-α) levels at three days are statistically associated with early LFCN injury, showing statistical significance (*P* < 0.05), as shown in Table 9.

**Table 8:** Analysis of differences in general conditions and demographics, between the early LFCN injury and No LFCN injury groups.

Factors		LFCN Injury group (*n* = 24)	NO LFCN Injury group (*n* = 76)	χ2/t	*P* value
Gender (female/male)		12/12	38/38	0.000	1.000
Age (years)		63.07 ± 9.64	63.50 ± 10.56	−0.177	0.853
BMI		24.65 ± 3.08	25.87 ± 4.84	−1.161	0.154
Underweight (BMI <18.5 kg/㎡)		0	2		
Normal weight (18.5 kg/m ≤ BMI <24.0 kg/m^2^)		10	23	2.177	0.536
Overweight (24.0 kg/m ≤ BMI <28.0 kg/m)		9	39		
Obese (≥28.0 kg/㎡)		3	12		
ASA classification		2.04 ± 0.59	1.96 ± 0.61	0.564	0.568
Surgery duration(min)		13.82 ± 10.78	12.68 ± 1.94	0.887	0.613
Surgical incision length(cm)		7.37 ± 0.52	7.48 ± 0.45	−1.005	0.739
Intraoperative blood loss		118.04 ± 9.29	122.00 ± 31.21	−0.611	0.575
Surgery duration		80.79 ± 8.53	78.08 ± 7.89	1.439	0.175
Hemoglobin concentration loss 1 day after surgery		1.95 ± 0.43	1.94 ± 0.43	0.099	0.979
Disease diagnosis	Osteoarthritis	7	26		
	Femoral head necrosis	8	25		
	Congenital hip dislocation	2	7	0.376	0.945
	Femoral neck fracture	7	18		
	Preoperative	118.99 ± 9.26	114.48 ± 9.22	2.087	**0**.**042**

Statistically significant differences are indicated by bold *p*-values.

**Table 9:** Analysis of differences in postoperative inflammatory levels between the early LFCN injury and No LFCN injury groups.

Factors		LFCN injury group (*n* = 24)	NO LFCN injury group (*n* = 76)	χ2/t	*P* value
CK (U/L)	Immediately postoperative	223.53 ± 13.35	228.41 ± 10.94	−1.804	0.110
	3 days postoperative	343.79 ± 23.85	345.68 ± 21.28	−0.368	0.733
	Preoperative	4.91 ± 2.94	4.65 ± 2.74	0.398	0.704
CPR (mg/L)	Immediately postoperative	74.25 ± 29.95	74.68 ± 27.11	−0.066	0.951
	3 days postoperative	59.66 ± 29.95	60.62 ± 24.64	0.158	0.889
	Preoperative	101.71 ± 21.03	101.38 ± 22.62	0.063	0.947
IL-6 (pg/ml)	Immediately postoperative	172.56 ± 27.36	183.10 ± 29.56	−1.549	0.114
	3 days postoperative	310.64 ± 52.14	371.48 ± 107.14	−2.677	**<0**.**001**
	Preoperative	27.30 ± 3.43	27.68 ± 3.42	−0.474	0.641
IL-1β (pg/ml)	Immediately postoperative	47.92 ± 5.49	48.03 ± 5.13	−0.090	0.932
	3 days postoperative	72.78 ± 7.14	75.49 ± 9.53	−1.282	0.144
	Preoperative	25.80 ± 14.68	20.82 ± 13.25	1.564	0.145
TNF-α (pg/L)	Immediately postoperative	63.97 ± 21.87	63.63 ± 20.94	0.069	0.948
	3 days postoperative	77.01 ± 21.97	90.85 ± 34.31	−1.856	0.024

Statistically significant differences are indicated by bold *p*-values.

After a one-year follow-up, this study analyzed the recovery of LFCN and identified six cases of persistent LFCN injury. Similar analysis was conducted regarding potential risk factors, revealing that factors such as age, gender, disease diagnosis, surgery duration, ASA classification, incision length, intraoperative blood loss, postoperative drainage volume, and postoperative day 1 hemoglobin concentration did not show significant statistical significance (*P* > 0.05) in relation to persistent LFCN injury following DAA total hip arthroplasty. However, diabetes, preoperative serum CK levels, postoperative day 3 serum IL-6 levels, and postoperative day 3 serum TNF-α levels were found to be statistically significant (*P* < 0.05) in association with persistent LFCN injury, as shown in Table 10.

**Table 10:** Analysis of differences in general conditions, demographics, and postoperative inflammatory levels between the early LFCN injury and no LFCN injury groups.

Factors		LFCN injury group (*n* = 6)	NO LFCNinjury Group (*n* = 94)	χ2/t	*P* value
Gender (female/male)		2/4	48/46	0.709	0.400
Age (years)		68.50 ± 5.38	62.83 ± 9.95	1.378	0.176
BMI (kg/m^2^)		29.57 ± 4.66	24.65 ± 3.32	3.435	**0**.**001**
Smoking history (yes/no)		1/5	17/77	0.008	0.930
Alcohol consumption history (yes/no)		1/5	25/69	0.289	0.591
Hypertension (yes/no)		2/4	21/73	0.385	0.535
Diabetes (yes/no)		3/3	10/84	7.726	**0**.**005**
Hyperlipidemia (yes/no)		0/6	6/88	-	1.000
Underweight(BMI <18.5 kg/㎡)		0	2		
Normal weight(18.5 kg/m ≤ BMI <24.0 kg/m^2^)		2	31		
Overweight (24.0 kg/m ≤ BMI <28.0 kg/m)		2	46	1.416	0.709
Obese (≥28.0 kg/㎡)		2	15		
ASA classification		2.00 ± 0.82	2.02 ± 0.59	−0.079	0.934
Surgery duration (min)		83.83 ± 7.06	79.90 ± 8.45	1.113	0.275
Surgical incision length (cm)		7.62 ± 0.51	7.38 ± 0.50	1.139	0.276
Intraoperative blood loss		124.17 ± 19.68	118.66 ± 30.16	0.440	0.665
Surgery duration		83.83 ± 7.06	79.90 ± 8.45	1.113	0.275
Hemoglobin concentration loss 1 day after surgery		2.20 ± 0.40	1.93 ± 0.43	1.496	0.140
Disease Diagnosis	Osteoarthritis	1	32		
	Femoral head necrosis	1	32	6.039	0.110
	Congenital hip dislocation	0	9		
	Femoral neck fracture	4	21		
	Preoperative	118.10 ± 8.20	117.90 ± 9.49	0.050	0.960
CK (U/L)	Immediately postoperative	227.33 ± 12.02	224.54 ± 13.20	0.504	0.613
	3 days postoperative	344.12 ± 23.55	346.20 ± 16.96	0.210	0.834
	Preoperative	4.12 ± 1.61	4.89 ± 2.95	−0.631	0.531
CPR (mg/L)	Immediately postoperative	84.40 ± 22.40	73.71 ± 29.68	0.865	0.391
	3 days postoperative	71.82 ± 21.23	59.13 ± 29.15	1.047	0.300
	Preoperative	103.35 ± 23.22	101.52 ± 21.27	0.203	0.841
IL-6 (pg/ml)	Immediately postoperative	184.45 ± 19.23	174.49 ± 28.50	0.842	0.408
	3 days postoperative	463.78 ± 149.46	316.40 ± 57.21	5.372	**<0**.**001**
	Preoperative	29.38 ± 3.52	27.26 ± 3.39	1.482	0.145
IL-1β (pg/ml)	Immediately postoperative	50.30 ± 7.02	47.80 ± 5.26	1.107	0.276
	3 days postoperative	79.35 ± 16.31	73.05 ± 6.79	1.976	0.058
	Preoperative	25.87 ± 12.49	24.52 ± 14.55	0.222	0.828
TNF-α (pg/L)	Immediately postoperative	65.35 ± 25.63	63.79 ± 21.28	0.172	0.866
	3 days postoperative	112.65 ± 44.74	78.27 ± 24.48	3.152	**0**.**002**

Statistically significant differences are indicated by bold *p*-values.

### 3.9 Multifactorial analysis of risk factors for persistent LFCN injury

Multifactorial binary logistic regression analysis indicates that whether the limb is on the left or right side and whether the tensor fasciae latae muscle is injured are not significantly associated with the occurrence of postoperative LFCN complications (*P* > 0.05). However, diabetes, patient body mass index (BMI), IL-6 levels in peripheral blood 3 days after surgery, and TNF-α levels in peripheral blood 3 days after surgery are significantly associated with the occurrence of postoperative LFCN complications (*P* < 0.05), as shown in Table 11.

**Table 11:** Logistic regression analysis of factors associated with postoperative LFCN injury.

Factors	Regression coefficient	Standard error	OR value	95%CI
Diabetes	1.145	1.119	3.143	0.2810–28.15
BMI	0.2276	0.1287	1.256	0.9864–1.668
Serum IL-6 level 3 days post-op (pg/ml)	0.011	0.006	1.011	1.001–1.026
Serum TNF-α level 3 days post-op (pg/L)	0.031	0.016	1.032	1.001–1.068

### 3.10 Prediction model construction for LFCN injury

**Figure 2:**
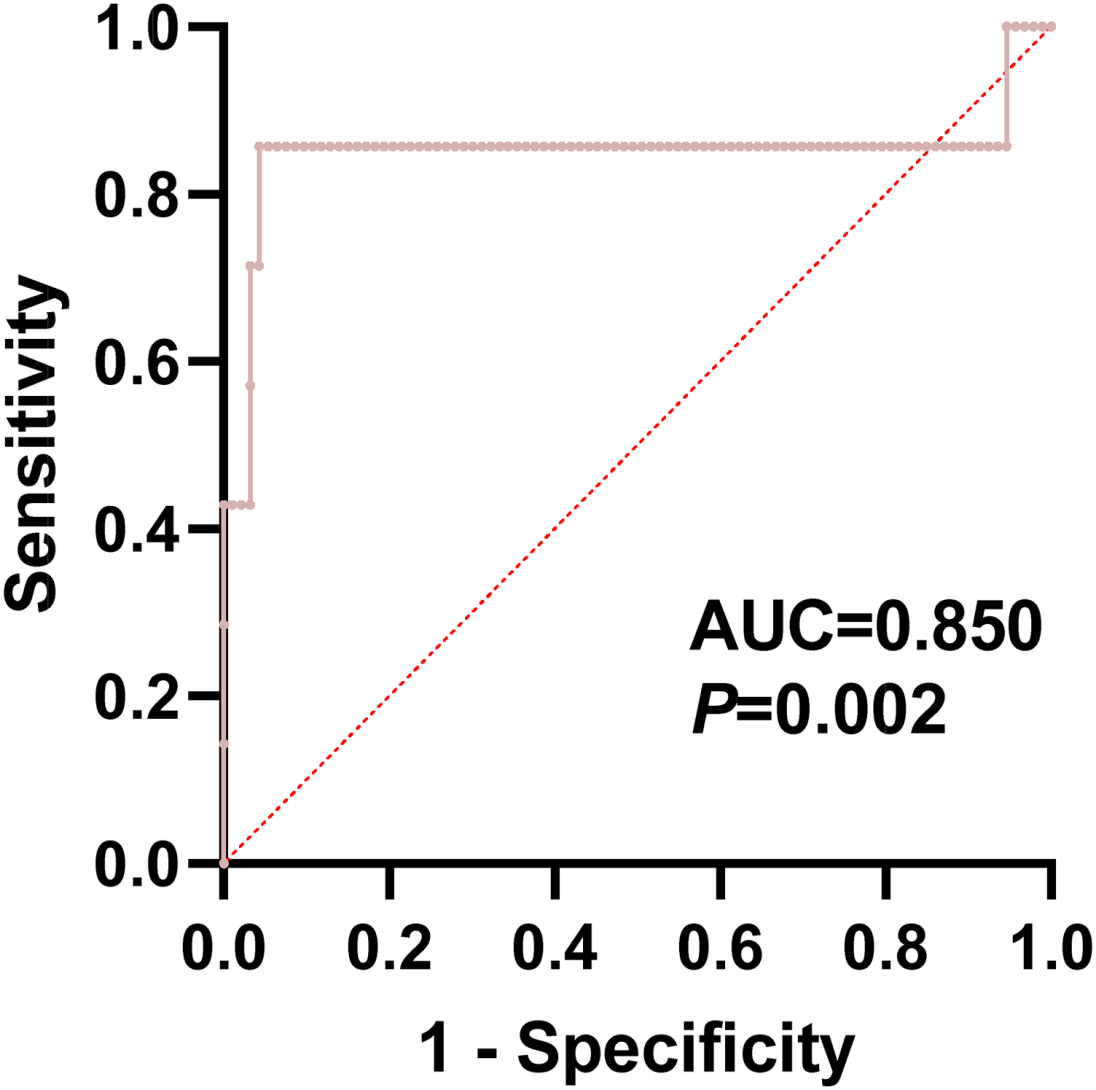
ROC curve for the occurrence of LFCN injury after DAA total Hip arthroplasty.

A multiple logistic regression analysis revealed that diabetes, BMI, serum IL-6 level 3 days post-op, and serum TNF-α level 3 days post-op are optimal indicators for predicting LFCN injury in patients undergoing DAA total hip arthroplasty. Based on these four indicators, a prediction model was constructed, and an ROC curve was plotted using Graphpad software, with an AUC of 0.850 (95% CI, 0.607–1.000, *P* = 0.0021). The prediction model demonstrated good discriminative ability, with a negative predictive value of 95.88% and a positive predictive value of 100.00%, indicating high accuracy and reliability in predicting LFCN injury in patients, as shown in Figure 2.

## 4 Discussion

When discussing the application value of Direct Anterior Approach (DAA) and Posterior Lateral Approach (PLA) in total hip arthroplasty, this study provides some key insights. Firstly, the DAA group demonstrated a series of advantages in postoperative recovery compared to the PLA group, including reduced incision length, decreased intraoperative blood loss and postoperative drainage, as well as significantly shortened hospital stay (19). These results suggest that the DAA technique may offer a more efficient surgical approach, aiding in reducing patients’ postoperative recovery time and potential risks of postoperative complications (20).

Although there were no significant differences in serum biomarkers (such as CK, CRP, IL-6, IL-1β, and TNF-α) (21) between the two groups in the early postoperative period, the levels of these indicators in the DAA group were significantly lower than those in the PLA group at 3 days postoperatively. This may reflect a smaller inflammatory response induced by the DAA method compared to the PLA method, which may promote faster postoperative recovery. Additionally, the VAS pain scores in the DAA group at 24 h, 48 h, and 72 h postoperatively were significantly better than those in the PLA group, further supporting the potential advantages of the DAA technique in alleviating postoperative pain.

Regarding the recovery of hip joint function, although both groups showed significant improvement in Harris scores postoperatively, the Harris scores in the DAA group were significantly higher than those in the PLA group at 2 weeks and 4 weeks postoperatively, indicating that the DAA technique may be more beneficial for short-term recovery of hip joint function. However, it is noteworthy that this advantage was no longer apparent at 6 months postoperatively, suggesting that there may be no significant difference between DAA and PLA techniques in long-term hip joint function recovery.

However, the DAA technique also has potential drawbacks, particularly a higher risk of LFCN injury (22, 23). This study showed that the incidence of LFCN injury in the DAA group was significantly higher than that in the PLA group. This issue not only increases postoperative discomfort but may also have adverse effects on patients’ long-term satisfaction and quality of life (24, 25). This finding suggests that although the DAA technique performs well in many aspects, special attention is needed during surgical planning and execution to avoid nerve injury. The surgical team should remain highly vigilant about the risk of LFCN injury and take corresponding preventive measures (26–28).

This study also further explored the independent risk factors for LFCN injury and found that diabetes, patient body mass index (BMI), and early postoperative levels of IL-6 and TNF-α were closely related to LFCN injury. This finding provides important evidence for clinicians to assess the risk of LFCN injury in patients preoperatively. By identifying high-risk patients early, physicians can proceed with surgery more cautiously or adopt other strategies to reduce the likelihood of LFCN injury.

The Harris Hip Score is a widely used assessment tool to quantify the functional recovery of patients after hip replacement (29). This scoring system comprehensively evaluates the functional status of the hip joint in six aspects: pain, function, mobility, muscle strength, deformity, and stability, providing an objective and quantifiable evaluation index for clinicians and patients (30). In this study, we used the Harris Hip Score to assess the functional recovery of the hip joint in patients after two types of total hip arthroplasty (THA), DAA (direct anterior approach) and PLA (posterior lateral approach). The results showed that Harris scores were significantly higher in the DAA group than in the PLA group at 2 and 4 weeks postoperatively, suggesting that the DAA technique is more conducive to the recovery of hip function in the short term. This finding is consistent with previous studies and further validates the advantages of the DAA technique in total hip arthroplasty.

It is worth noting that the risk prediction model established in this study has high accuracy, with an area under the receiver operating characteristic curve (AUC) of 0.850, indicating good discriminative ability. The model's high sensitivity and specificity demonstrate its reliability in predicting LFCN injury; the application of this prediction model can not only guide clinicians in making more personalized decisions in surgical planning and patient management but also provide patients with more accurate expectations of postoperative recovery, thereby optimizing the overall treatment experience (31).

In summary, although DAA-THA demonstrates advantages in reducing perioperative bleeding, alleviating postoperative pain, and promoting rapid recovery, its high risk of LFCN injury cannot be ignored (32). By gaining a deeper understanding of risk factors and applying risk prediction models, surgical teams can better assess and prepare for LFCN injury risk preoperatively to minimize the risk of LFCN injury and improve patients’ postoperative satisfaction and quality of life (33). Future research should continue to explore improvements in surgical techniques and strategies to further enhance the safety and effectiveness of THA. Additionally, with the continuous advancement of surgical techniques and surgeons’ experience, these preliminary findings may change, requiring more research to validate and expand our results.

Our study focuses on the effects of direct anterior total hip arthroplasty (DAA-THA) vs. posterolateral approach (PLA) on ilioinguinal cutaneous nerve (LFCN) injury in total hip arthroplasty. In the article, we not only compared the differences in the incidence of LFCN injury between the two surgical approaches but also analysed the risk factors for LFCN injury in depth and constructed a prediction model, which is less addressed in other studies.

While the results of this study provide important insights into the application of DAA and PLA approaches in THA, it is important to recognize the limitations of the study, including a small sample size and short follow-up time. The limited sample size and short follow-up time, along with subjective reporting scores and difficulty in excluding other influencing factors, may limit the reliability of the conclusions. Future studies can build on this foundation by expanding the sample size, extending the follow-up period, and incorporating more objective indicators for validation. Considering the economic feasibility and clinical minimally invasive nature of this surgical approach, we believe that the conduct of this study will bring new thinking to the conduct of total hip arthroplasty surgery and is expected to improve the current diagnosis and treatment status of related diseases.

## 5 Conclusion

In this study, by comparing direct anterior approach (DAA) with posterior approach (PLA) hip arthroplasty, we found that the DAA group had potential advantages in postoperative recovery, but was accompanied by a higher risk of lateral femoral cutaneous nerve (LFCN) injury. The study constructed a prediction model for LFCN injury, which demonstrated good discriminative ability with an AUC of 0.850 and may provide a powerful tool for clinical decision-making. Although DAA-THA demonstrates the potential to promote rapid recovery, its application requires careful consideration of individual patient differences and potential risks. Therefore, it is recommended that a comprehensive preoperative assessment of the patient's condition be performed, combined with the results of the prediction model, to formulate a personalised surgical plan that balances surgical efficacy and safety.

## Data Availability

The original contributions presented in the study are included in the article/Supplementary Material, further inquiries can be directed to the corresponding author.
